# Short term real-world effectiveness of faricimab in neovascular age-related macular degeneration patients in the Republic of Korea

**DOI:** 10.1038/s41598-026-41488-1

**Published:** 2026-03-05

**Authors:** Doh Hoon Chung, Donghyun Jee, Young Jung Roh, Ho Ra, Mee Yon Lee, Jae Yon Won, Jeong Ah Shin, Suk Hoon Jung, Young-Hoon Park

**Affiliations:** 1 Department of Ophthalmology and Visual Science, College of Medicine, The Catholic University of Korea, Seoul, Republic of Korea; 2 Department of Ophthalmology and Visual Science, Seoul St. Mary’s Hospital, College of Medicine, The Catholic University of Korea, Seoul, Republic of Korea; 3 Department of Ophthalmology and Visual Science, Yeouido St. Mary’s Hospital, College of Medicine, The Catholic University of Korea, Seoul, Republic of Korea; 4 Department of Ophthalmology and Visual Science, Bucheon St. Mary’s Hospital, College of Medicine, The Catholic University of Korea, Bucheon, Gyeonggi-do Republic of Korea; 5 Department of Ophthalmology and Visual Science, College of Medicine, St. Vincent’s Hospital, The Catholic University of Korea, Suwon, Republic of Korea; 6 Department of Ophthalmology and Visual Science, Uijeongbu St. Mary’s Hospital, College of Medicine, The Catholic University of Korea, Uijeongbu- si, Republic of Korea; 7 Department of Ophthalmology and Visual Science, Eunpyeong St. Mary’s Hospital, College of Medicine, The Catholic University of Korea, Seoul, Republic of Korea; 8 Department of Ophthalmology and Visual Science, Daejeon St. Mary’s Hospital, College of Medicine, The Catholic University of Korea, Seoul, Republic of Korea; 9 Department of Ophthalmology and Visual Science, Incheon St. Mary’s Hospital, College of Medicine, The Catholic University of Korea, Seoul, Republic of Korea

**Keywords:** Diseases, Health care, Medical research

## Abstract

To analyze visual and anatomic outcomes of faricimab injection in neovascular age-related macular degeneration (nAMD) in a real-world setting in Korea. We collected data from nAMD patients who received faricimab injection from 2024 to 2025. The past injection history, and faricimab injection history was obtained. Visual acuity (VA) and central macular thickness (CMT) outcomes for 1, 3, 6, 12 months after initial faricimab injection were compared to baseline, and also compared between sex, different age-groups, and naïve vs. non-naïve groups. A total of 286 patients, 293 eyes from 8 university hospitals were included in this study. VA improved from baseline 58.9 ± 17.1 letters to 60.7 ± 23.5, 61.0 ± 18.1, 59.9 ± 19.3, 60.5 ± 18.2 letters at 1, 3, 6, 12 months respectively (statistically significant at 3 months, *p* = 0.009), while CMT improved by -60.6 ± 89.0, -55.9 ± 87.3, -46.5 ± 97.5, -70.7 ± 97.8 μm compared to baseline (*p* < 0.001 at all timepoints). An age group analysis showed that the youngest age group (< 60 years) had superior VA results, while the naïve group showed superior outcomes compared to the non-naïve group. CMT fluctuation showed inverse correlation with visual acuity at 6 months, with lower CMT fluctuation being associated with the young and naïve. Faricimab injection for nAMD, in a real-world setting in the Republic of Korea, showed significant functional and anatomic improvements with superior results in the younger age group and naïve group, suggesting promising effectiveness of a bispecific blockage of VEGF-A and Ang-2 at the initial phases of nAMD.

## Introduction

Age-related macular degeneration (AMD) is a leading cause of irreversible vision loss among older adults worldwide, with neovascular AMD (nAMD) and geographic atrophy (GA) contributing to substantial, and progressive visual impairment^1,2^. The treatment of GA (an advanced form of dry AMD) has emerged only recently with promising effects in slowing the growth of geographic lesions, but with limited improvement in visual function^3^. On the other hand, the emergence of intravitreal anti-vascular endothelial growth factor (anti-VEGF) therapy has dramatically improved the visual prognosis of nAMD patients in the past 20 years, making anti-VEGF injections the standard treatment for nAMD^4^. Despite these advances in nAMD treatment, many patients still require frequent injections and long-term treatment, posing a considerable burden on both patients and the health-care system^5^.

Of the anti-VEGF treatments, faricimab is the first bispecific antibody that simultaneously targets VEGF-A and angiopoietin-2 (Ang-2). By addressing two different pathways involved in vascular instability and neovascularization, faricimab has the potential to achieve improved durability and anatomic outcome compared to conventional anti-VEGF mono-therapies^6^. Pivotal clinical trials, including the TENAYA and LUCERNE studies, demonstrated that faricimab provides non-inferior visual outcomes to aflibercept while enabling extended treatment intervals up to 16 weeks in a significant proportion of patients^7^. These results have generated significant expectations in reducing the treatment burden and improving long-term outcome in patients with nAMD.

However, evidence supporting faricimab use in nAMD has largely been derived from randomized clinical trials (RCTs), which may not fully reflect real-world clinical settings, variability in treatment adherence, and heterogenous patient characteristics^8^. In this regard, although several studies reported real-world experience of faricimab use in nAMD patients^9–12^, there remains limited real-world data among the Korean population. Different confounding factors compared to cohorts of other real-world studies – such as the fact that nAMD with polypoidal choroidal vasculopathy is more prevalent in the Asian and Korean population^13,14^– may show different real-world clinical response of nAMD to faricimab.

Therefore, this study aims to evaluate the real-world efficacy and durability of faricimab in Korean patients with nAMD. By analyzing treatment outcomes of eight university hospitals (using the Catholic Medical Center Big Data Integration Center), we seek to provide clinically relevant insights that complement existing RCT evidence and help guide optimum treatment strategies for nAMD.

## Materials and methods

### Data source

Data from January 2024 to August 2025 were extracted in 2025 from the Clinical Data Warehouse (CDW) of the Catholic University Big Data Integration Center (https://cohort.cmcnu.or.kr.). The original data and electronic medical records were extracted from eight university hospitals of the Catholic University of Korea (Bucheon St. Mary’s Hospital, Daejeon St. Mary’s Hospital, Eunpyeong St. Mary’s Hospital, Incheon St. Mary’s Hospital, Yeouido St. Mary’s Hospital, Uijeongbu St. Mary’s Hospital, St. Vincent’s Hospital, and Seoul St. Mary’s Hospital). The data of the patient’s age, sex, diagnosis, past history of anti-VEGF treatments, date of initial faricimab injection, date of visit, best-corrected VA, and OCT images were extracted from the records. Patient identifiers, treatment sites, and clinician data were removed and anonymized. All methods were performed in accordance with the relevant guidelines and regulations; the study adhered to the tenets of the Declaration of Helsinki, and all protocols were approved by the Institutional Review Board/Ethics Committee of Catholic Medical Center (Republic of Korea) (2025-1030-0001). As this was a retrospective study with anonymized data, requirements for informed consent were waived by the Institutional Review Board/Ethics Committee of Catholic Medical Center (Republic of Korea).

### Study design

This was a retrospective, single-arm, multicenter, non-randomized cohort study of visual and anatomic outcomes in patients with nAMD treated with faricimab. Data from January 2024 to August 2025 was extracted in 2025. Patients aged ≥ 55years with a diagnosis code of nAMD (Korean Classification of Disease Code H3135) and a concomitant procedure code for the intravitreal injection of faricimab were searched from the CDW database. All patients were observed for a minimum of 3 months and received at least one faricimab injection during the study period. The index date was defined as the date of the first faricimab injection. The treatment protocol of faricimab injection was 3 monthly injections followed by a treat and extend protocol. However, as expected in a real-world setting, pro re nata injections were performed in a number of patients due to treatment adherence issues or socioeconomic situations. In patients who switched to another anti-VEGF agent after a suboptimal response to faricimab, the VA, CMT results were excluded from there onwards – i.e. the VA, CMT data just until the decision of switching were included in the study. Injections administered for diagnostic purposes or response assessment in cases of drusenoid pigment epithelial detachment or central serous chorioretinopathy were not included. Patients who had both eyes treated with faricimab had each eye to be separately included and analyzed in this study.

As the study involved eight different university hospitals, three different optical coherence tomography (OCT)s were used; two swept-source OCTs – Spectralis OCT (Heidelberg Engineering, Heidelberg, Germany), and Cirrus HD-OCT (Carl Zeiss Maditec, Dublin, CA, USA) – and one spectral domain OCT – DRI OCT Triton (Topcon Co., Tokyo, Japan). Many previous studies have shown the need for calibration when using different OCTs within a single study; some have argued that different OCTs cannot be used interchangeably in clinical monitoring^15–17^. The requirement for the calibration of the different OCTs wasn’t needed for this study, as each individual subject received the same OCT examination from baseline to each follow-timepoints. For statistical analysis, the change in CMT compared to baseline (rather than the absolute value of CMT) was obtained (named ΔCMT), thus minimizing the limitations of using three different OCTs in a single study.

### Study outcomes

The clinical outcomes were the mean VA and mean change in CMT (ΔCMT) at 1, 3, 6, 12 months after initial faricimab injection. All best-corrected VA measurements from the CDW were converted to approximate Early Treatment of Diabetic Retinopathy Study letter scores using previously established guidelines. From the 3 different OCTs, the distance between the vitreoretinal surface and the retinal pigment epithelium (RPE) was measured to obtain the CMT, given by the value at the center of the central 1 mm circle of the ETDRS grid in the OCTs. The imaging protocol used by each device were as follows; Spectralis OCT, volume scan that covered 30º×30º, 6 × 6 mm centered on the fovea; Cirrus HD-OCT, 6 × 6 mm with 512 × 128 macular cube; DRI OCT, 6 × 6 mm 3D macular scan. The difference in anatomical basis for thickness measurements in different OCTs have been previously described^16^; Spectralis OCT measures the distance from the internal limiting membrane (ILM) to the Bruch’s membrane (BM), while Cirrus HD-OCT measures from the ILM to the middle layer of the retinal pigment epithelium (RPE), and DRI OCT measures from the ILM to the border of the outer photoreceptor segment (OS) and RPE. To minimize the variability from using different OCTs, the CMT of every individual subject at each time point was subtracted by their baseline CMT (ΔCMT), which was then used for statistical analysis. Statistical analyses were conducted using IBM SPSS statistics, version 26.0 (IBM Corp., Armonk, NY, USA). Significance values were identified using two tailed *t*-test and One-way ANOVA, with statistically significant values were defined as *p* < 0.05.

### Sub-group analysis

Three sub-group analysis were conducted for this study. Firstly, the cohort was divided in to male and female subjects, with which an independent samples *t*-test was conducted for comparison of VA and CMT at each timepoint (intergroup analysis). A comparison of pre- and post- treatment outcomes within the same group (intragroup analysis) was performed using paired *t*-test. Secondly, the cohort was divided in to different age groups. With the index date defined as the date of first faricimab injection, the age groups were divided into 50s (≥ 55 and < 60 years), 60s (≥ 60 and < 70 years), 70s (≥ 70 and < 80 years), 80s (≥ 80 and < 90 years), 90s (≥ 90 years); One-way ANOVA was conducted for comparison of VA and CMT between the groups (intergroup analysis). Paired t-test was performed for comparison of pre- and post- treatment outcomes for the 60s, 70s and 80s age groups (intra group analysis); Wilcoxon signed rank test was performed for the 50s and 90s age group, due to a small sample size. Lastly, the cohort was divided in to naïve and non-naïve groups; an independent samples *t*-test was conducted to compare the VA and CMT (intergroup analysis), while paired t-test was performed to compare pre- and post- treatment outcomes within the same group (intragroup analysis).

### CMT fluctuation

As described in previous studies^18,19^, fluctuation in CMT during nAMD treatment with anti-VEGF injections can impact visual prognosis, with the two having an inverse correlation. To examine the effect of CMT fluctuation in the current study, standard deviation of CMT was obtained (CMT SD). A multiple linear regression analysis was performed to examine the change in VA at 6 months, with baseline, demographic and treatment characteristics, along with CMT SD as predictors. CMT SD was ranked in quartiles with which mean VA, and mean difference in VA was obtained to analyze its relationship with CMT fluctuation. To examine whether CMT fluctuation is affected by each subgroup, an additional multiple linear regression analysis was performed.

## Results

286 patients and 293 eyes were included in this study. Tables 1 and 2 summarizes the demographics and intravitreal injection history of the patients. The mean age at initial faricimab injection was 74.1 ± 8.5 with range [55–97 years]. There wasn’t a significant difference in sex (male vs. female) and eye treated. A total of 59 naïve eyes were included in the study which consisted 20% of the study population; 80% (234 eyes) of the patients were previously treated with a different anti-VEGF injection. The mean number of previous anti-VEGF injections was 12.45 ± 13.50, with a mean type of anti-VEGF used 1.82 ± 1.27. The mean interval of the previous injections was approximately 110.0 ± 107.4 days.

**Table 1:** Baseline demographic characteristics.

Number of patients	286	
Number of eyes	293	
	**Mean (SD)**	**Range**
Age	74.1 (8.5)	55–97
Age group	**Number**	**Percentage (%)**
55–59	17	6
60–69	76	26
70–79	123	42
80–89	71	24
90–99	6	2
Gender		
Male	167	57
Female	126	43
Eye treated		
Right	138	47
Left	155	53
Naïve	59	20
Presence of previous anti-VEGF injection	238	81

anti-VEGF: anti-vascular endothelial growth factor; SD: standard deviation.

**Table 2:** Anti-VEGF injection history.

Previous injections		
	Mean (SD)	
N. of previous anti-VEGFs used	1.8 (1.3)	
N. of previous anti-VEGF injections	12.5 (13.5)	
Mean interval of previous injections (days)	110.0 (107.4)	
**Faricimab injections**		
	**Mean (SD)**	
N. of faricimab injections	3.0 (1.7)	
Mean interval of faricimab (days)	70.3 (36.5)	
Follow-up time from baseline	188.3 (101.7)	
Type of OCT used	**Number**	**Percentage**
Topcon	181	62
Heidelberg	27	9
Cirrus	79	27
Insurance benefit for faricimab	241	82
Returned to other anti-VEGF after suboptimal results with Faricimab	85	29

anti-VEGF: anti-vascular endothelial growth factor; SD: standard deviation; N.: number; OCT: optical coherence tomography.

A mean of 3.03 ± 1.67 faricimab injections were performed with a mean interval of 70.3 ± 36.5 days. The mean follow-up time from baseline was a 186.9 ± 102.5 days. The different types of OCTs used were DRI OCT Triton (Topcon Co., Tokyo, Japan), Spectralis OCT (Heidelberg Engineering, Heidelberg, Germany), and Cirrus HD-OCT (Carl Zeiss Maditec, Dublin, CA, USA) which were used with a percentage of 62%, 9%, 27% respectively. 241 eyes were treated with faricimab with an insurance benefit, which consisted 82% of the treated eyes. In a total of 85 eyes (29%) returned or changed treatments to another anti-VEGF due to suboptimal results after faricimab treatment. This group with suboptimal response to faricimab had more frequent previous anti-VEGF injections – 15.9 ± 14.0 compared to 11.1 ± 13.1 injections of the group that continued faricimab injection, with statistically significant difference (*p* = 0.005) – and were predominantly of the non-naïve group (95.3%).

**Fig. 1:**
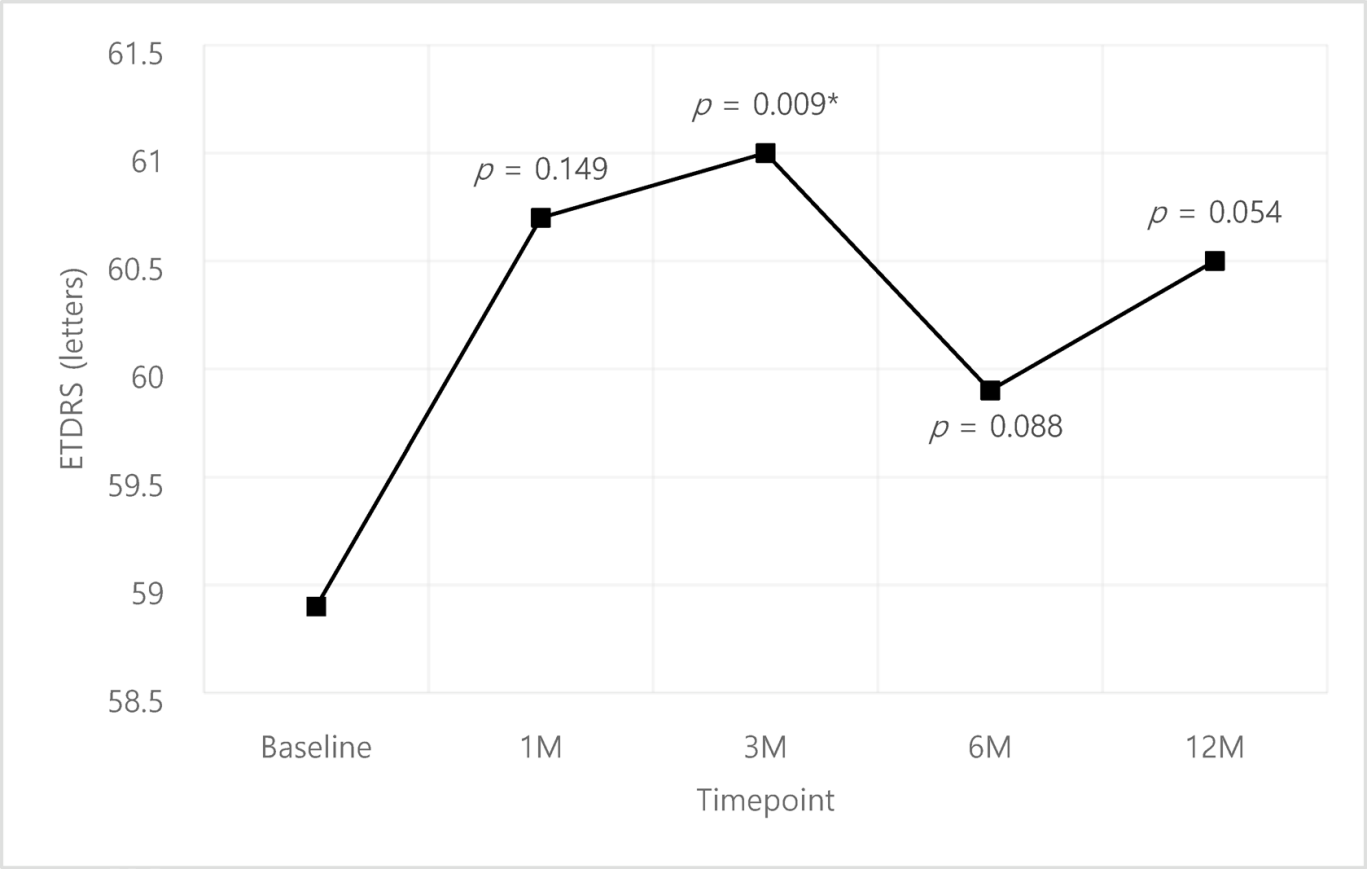
Overall visual acuity (ETDRS). Comparison of pre- and post-treatment values were analyzed using paired t-test.

**Fig. 2:**
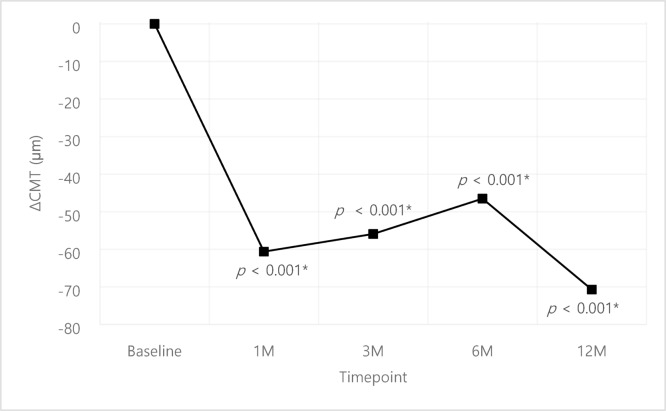
Overall change in Central macular thickness (µm). Comparison of pre- and post-treatment values were analyzed using paired t-test.

The VA results of the cohort is shown in Fig. 1. The mean VA at baseline was 58.9 ± 17.1 letters which improved to 60.7 ± 23.5, 61.0 ± 18.1, 59.9 ± 19.3 and 60.5 ± 18.2 at 1, 3, 6, 12 months after initial faricimab injection; statistically significant improvement was only identified at 3 months compared to baseline (*p* = 0.009). The CMT improved by -60.6 ± 89.0, -55.9 ± 87.3, -46.5 ± 97.5, -70.7 ± 97.8 μm compared to baseline at 1, 3, 6, 12 months respectively with statistically significant improvement at all time points (*p* < 0.001) (Fig. 2.).

**Fig. 3:**
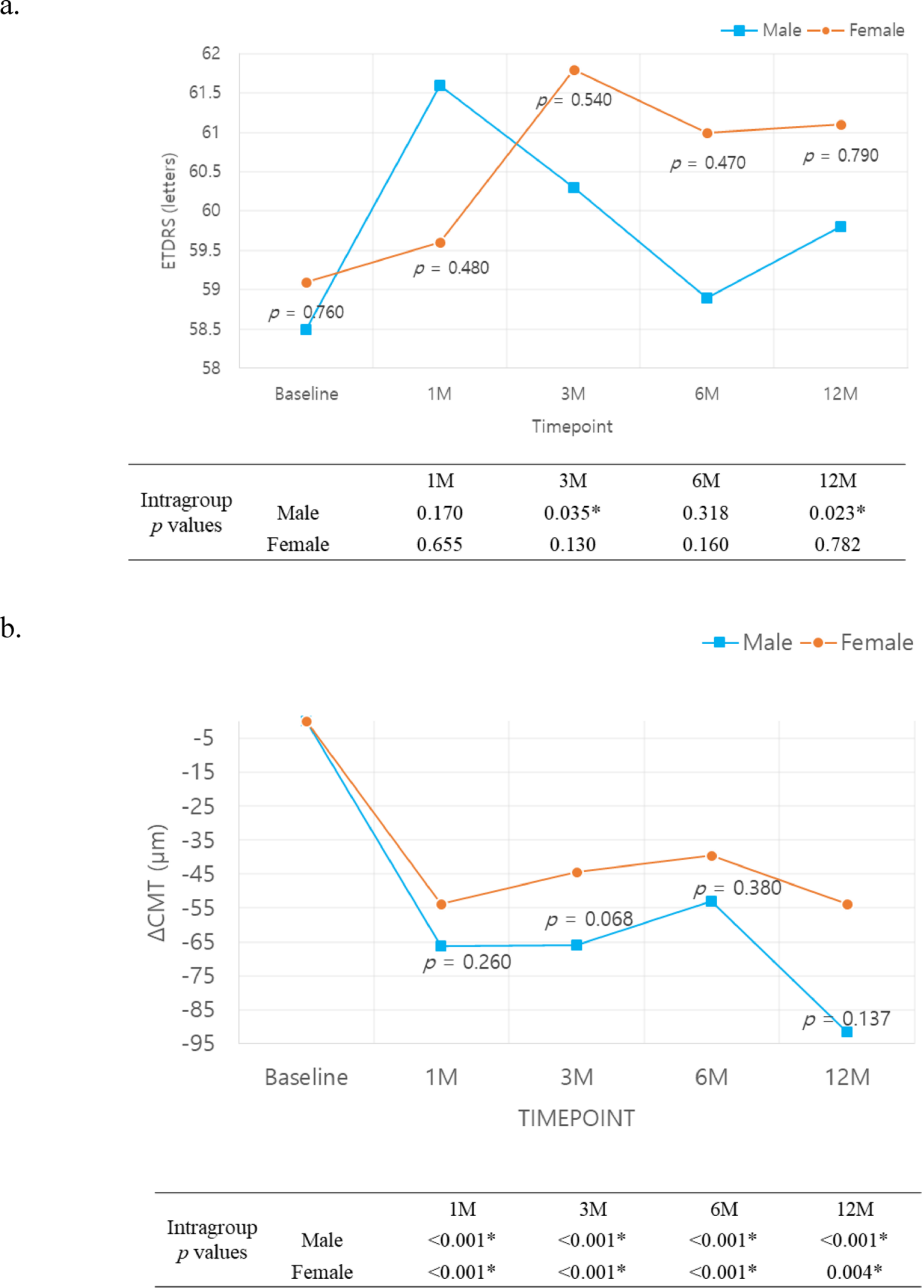
Visual acuity (**a**) and Central macular thickness (**b**) change by sex. *p*-values for intergroup analyses (independent samples *t*-test) are shown on each graph, while intragroup analyses (paired *t*-test) are depicted in the table below.

A subgroup analysis of the cohort of male vs. female showed no statistically significant difference in VA. Although CMT improvement was more evident in the male group, it lacked statistical significance (Fig. 3.).

**Fig. 4:**
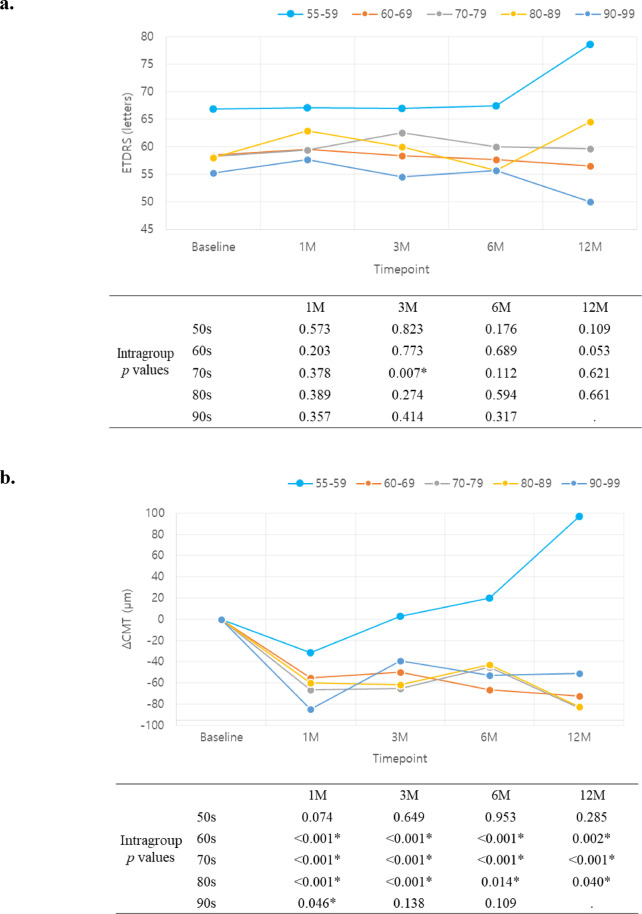
Visual acuity (**a**) and Central macular thickness (**b**) change by age group. *p*-values for intragroup analyses (paired *t*-test for 60s, 70s, 80s group and Wilcoxon signed rank test for 50s, 90s group) are depicted in the table below.

VA by age group showed no statistically significant difference at all timepoints; however, the youngest age group (50s) showed a tendency of superior VA compared to the other age groups with 67.2 ± 15.4, 67.0 ± 20.1, 67.5 ± 20.3, 78.7 ± 5.7 letters at 1, 3, 6, 12 months respectively (Fig. 4a). The youngest age group also showed a tendency of poorer CMT improvement, with CMT actually increasing at 3, 6, 12months (Fig. 4b.). However, it is worth noting that the baseline CMT of the 50s age group was thinner with 293.5 ± 97.6 μm, compared to 322.1 ± 82.2, 314.1 ± 92.7, 300.1 ± 95.1, 336.5 ± 62.5 μm of 60s, 70s, 80s, 90s age group, respectively.

**Fig. 5:**
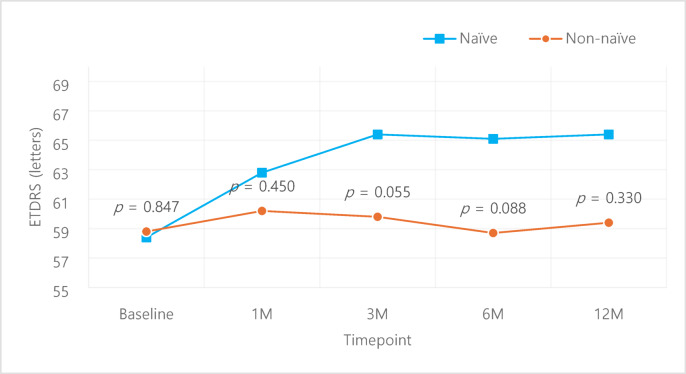
Visual acuity changes: Naïve vs. Non-naïve. *p*-values for intergroup comparison were analyzed using independent samples *t*-test.

**Fig. 6:**
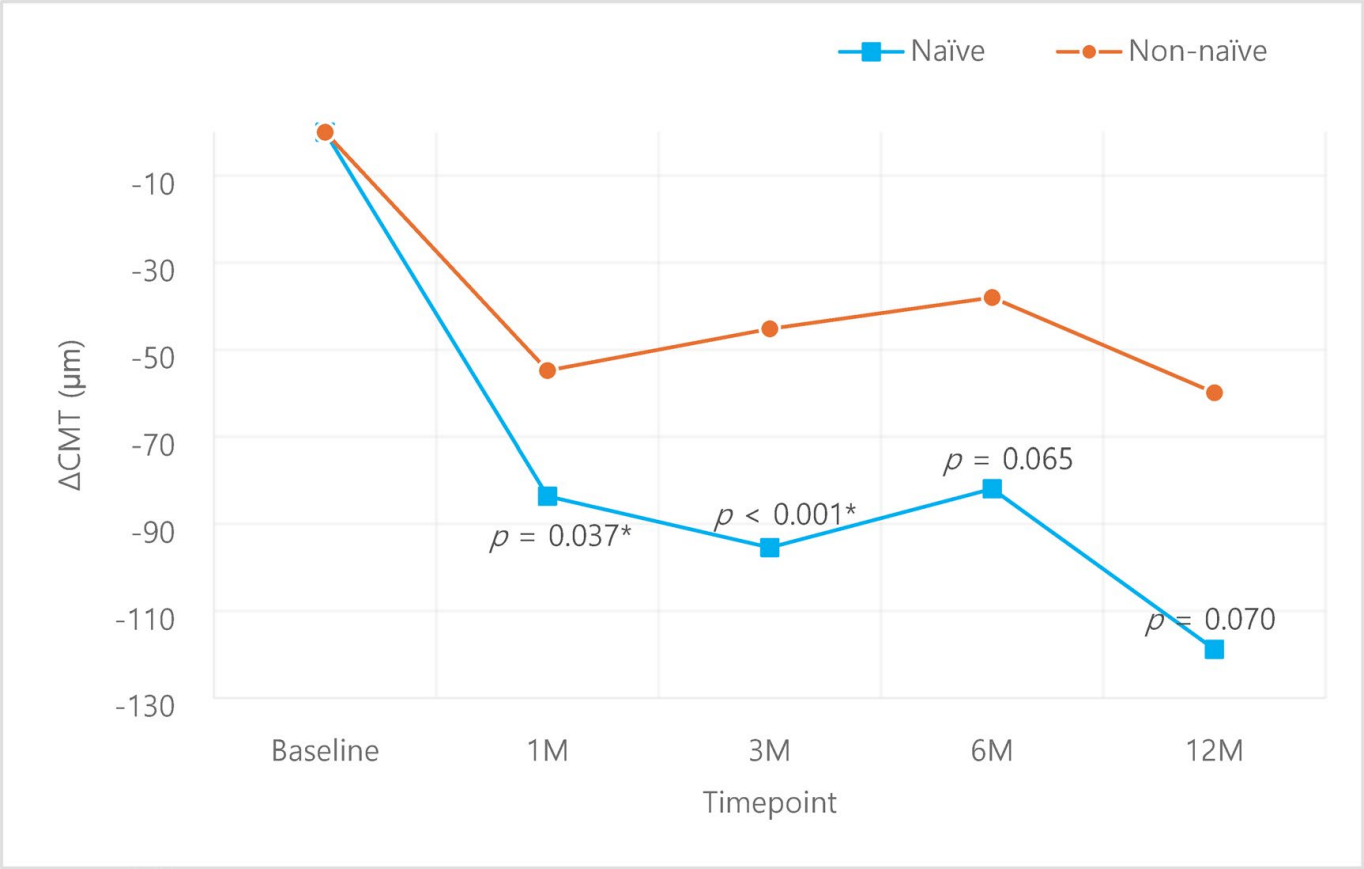
Central macular thickness changes: Naïve vs. Non-naïve. *p*-values for intergroup comparison were analyzed using independent samples *t*-test.

**Fig. 7:**
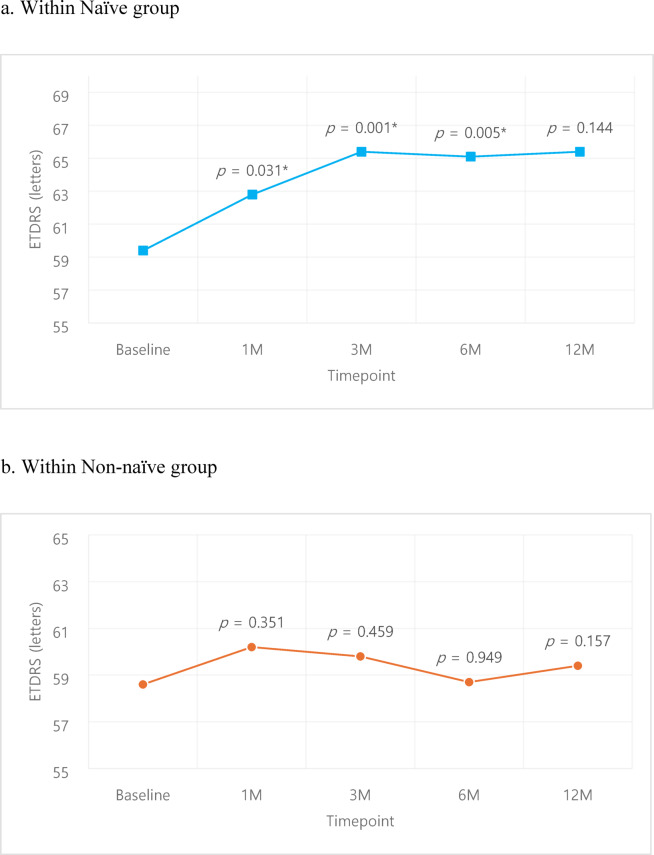
Visual acuity changes within each group: Naïve, Non-naïve. Comparison of pre- and post-treatment values were analyzed using paired *t*-test.

**Fig. 8:**
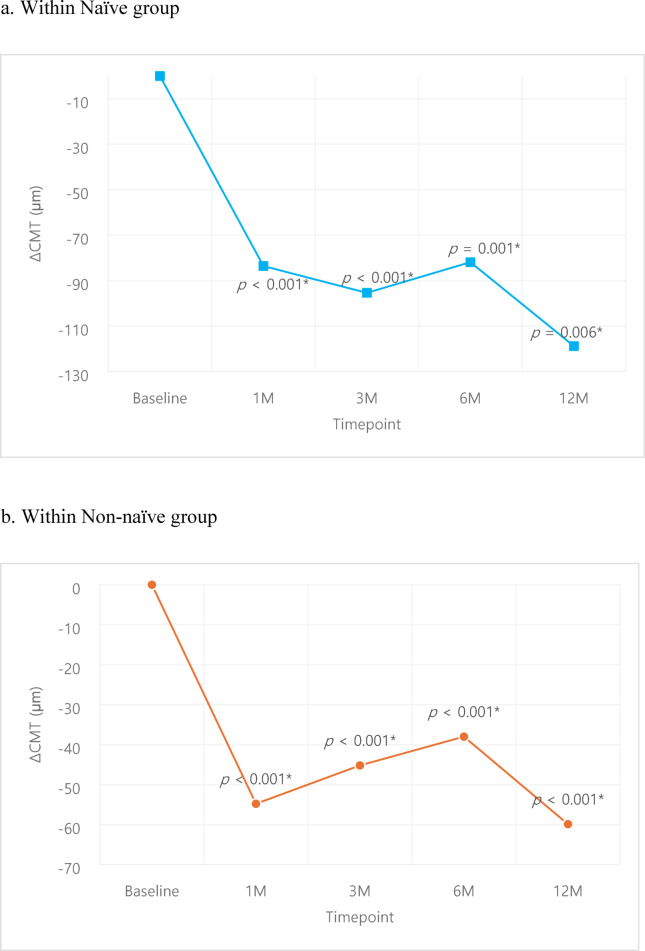
Central macular thickness changes within each group: Naïve, Non-naïve. Comparison of pre- and post-treatment values were analyzed using paired *t*-test.

A subgroup analysis of naïve vs. non-naïve patients showed superior outcomes of the naïve group in both VA and CMT. VA was better in the naïve group compared to the non-naïve group despite lacking statistical significance (Fig. 5.), while improvement in CMT was larger (Fig. 6) in the naïve group compared to the non-naïve group, with statistically significant superiority at 1, 3 months compared to the non-naïve group (*p* = 0.037, *p* < 0.001 respectively). Within the naïve group, VA improved from 58.4 ± 16.2 letters at baseline to 62.8 ± 15.4, 65.4 ± 15.1, 65.1 ± 15.9, 65.4 ± 12.7 letters at 1, 3, 6, 12 months respectively; statistical significance was noted at 1, 3, 6 months (*p* = 0.031, *p* = 0.001, *p* = 0.005 respectively) (Fig. 7a.). The non-naïve group showed VA improvement from baseline 58.8 ± 17.6 letters to 60.2 ± 25.2, 59.8 ± 18.7, 58.7 ± 19.9, 59.4 ± 19.2 letters at 1, 3, 6, 12 months respectively, although it lacked statistical significance (Fig. 7b.). Improvement in CMT compared to baseline was − 83.6 ± 91.3, -95.4 ± 84.0, -81.9 ± 123.4, -118.8 ± 114.1 μm 1, 3, 6, 12 months in the naïve group (Fig. 8a.), with statistical significance at all time points (*p* < 0.001, *p* < 0.001, *p* = 0.001, *p* = 0.006 respectively). CMT also improved compared to baseline by -54.8 ± 87.7, -45.2 ± 85.3, -38.0 ± 88.7, -59.9 ± 91.6 μm at 1, 3, 6, 12 months in the non-naïve group (Fig. 8b.), all with statistical significance (*p* < 0.001).

**Fig. 9:**
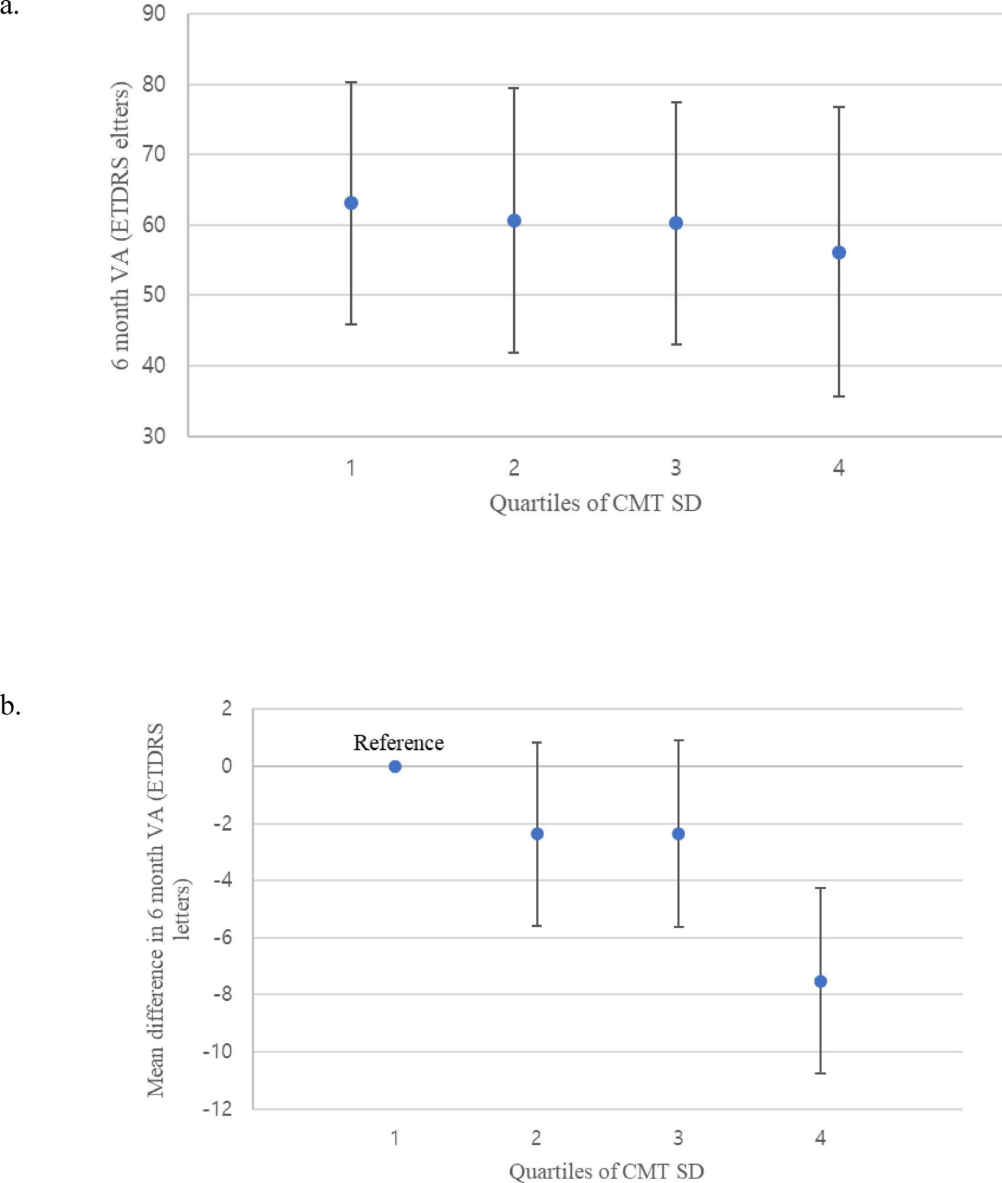
Mean 6 months visual acuity outcomes stratified by quartiles of CMT fluctuation (a) and mean difference in 6 months visual acuity (b) stratified by quartiles of CMT fluctuation, obtained by multiple linear regression, adjusted for baseline variables, demographics. Error bars in (**a**) represent the standard deviation and error bars in (**b**) represent the standard error at each quartile.

CMT fluctuation, as calculated by the standard deviation of CMT within each patient, showed an overall mean value of 51.9 ± 42.1 μm. Firstly, multiple linear regression was performed to asses the effect of predictor variables on VA at 6 months. As shown in Table 3., CMT fluctuation (-0.04 [-0.07, 0.00] ETDRS letters of 6 months VA per CMT SD, 0.036), along with baseline VA (0.83 [0.74, 0.91] ETDRS letters of 6 months VA per baseline VA, *p* < 0.001) and whether the patient was treatment-naïve (-5.20 [-9.21, -1.19] ETDRS letters of 6 months VA in non-naïve compared to naïve), appeared as statistically significant predictors of VA at 6 months. Age, systemic comorbidities (diabetes, hypertension), gender, number of previous anti-VEGF injections didn’t show significant association with VA at 6 months. To further examine the relationship between VA and CMT fluctuation, CMT SD were stratified in to quartiles. Quartiles 1, 2, 3, 4 showed 6 months VA result of 63.1 ± 17.2, 60.7 ± 18.8, 60.3 ± 17.2, 56.2 ± 20.6 respectively; there was a tendency of higher visual outcome with lower CMT fluctuation, despite lacking statistical significance (Fig. 9a.). The mean difference in 6 months VA obtained by a multiple linear regression model, with baseline variables and demographics as predictors (using the same predictors as in the model given in Table 3). Quartile 2 (-2.37 [-8.67, 3.92] ETDRS letters of 6 months VA, *p* = 0.459) and Quartile 3 (-2.35 [-8.76, 4.05] ETDRS letters of 6 months VA, *p* = 0.470) didn’t show statistically significant difference compared to Quartile 1, while Quartile 4 (-7.52 [-13.92, -1.12] ETDRS letters of 6 months VA, *p* = 0.021*) showed statistically significant difference compared to Quartile 1 (Fig. 9b.).

**Table 3:** Multiple linear regression model for change in VA at 6 month, using CMT SD as a predictor.

Factor	6 months Estimates	95% CI	*p* Value
(Intercept)	25.49	10.67 to 40.31	< 0.001*
Age, yr	-0.10	-0.28 to 0.07	0.255
DM (Reference: no DM)	-0.12	-3.92 to 3.69	0.952
HTN (Reference: no HTN)	0.01	-2.97 to 3.00	0.993
Baseline visual acuity, ETDRS letters	0.83	0.74 to 0.91	< 0.001*
CMT SD, µm	-0.04	-0.07 to -0.00	0.036*
Gender (Male)	0.35	-2.54 to 3.23	0.813
Number of previous anti-VEGF injections	-0.02	-0.14 to 0.10	0.698
Naïve vs. Non-naïve (Naïve)	-5.20	-9.21 to -1.19	0.011*
**Model characteristics**			
R²/adjusted R²	0.619/0.607		

CI: confidence interval; DM: Diabetes Mellitus; HTN: Hypertension; CMT: central macular thickness; SD: standard deviation; R²: coefficient of determination.

CMT fluctuation and its relationship with the subgroup analyzed above, was assessed using multiple linear regression (Table 4). Number of previous injections (-0.51 [-0.92, -0.10] µm of CMT SD per number of previous injections, *p* = 0.014*) appeared as a statistically significant predictor, while systemic comorbidities, age, gender, whether the patient was treatment-naïve, didn’t show statistically significant association.

**Table 4:** Multiple linear regression model for change in CMT variability (CMT SD).

Factor	6 months Estimates	95% CI	*p* Value
(Intercept)	36.62	-11.56 to 84.79	0.136
Age, yr	0.31	-0.31 to 0.93	0.997
DM	2.05	-11.29 to 15.38	0.763
HTN	-2.60	-13.15 to 7.96	0.628
Gender (Male)	-9.03	-19.12 to 1.06	0.079
Number of previous anti-VEGF injections	-0.51	-0.92 to -0.10	0.014*
Naïve vs. Non-naïve (Naïve)	3.60	-10.59 to 17.79	0.618
**Model characteristics**			
R²/adjusted R²	0.037/0.014		

CI: confidence interval; DM: Diabetes Mellitus; HTN: Hypertension; CMT: central macular thickness; R²: coefficient of determination.

## Discussion

This study conducted an analysis of 286 patients (293 eyes) from 8 different university hospitals, and is the first real-world study in Korea of nAMD patients treated with faricimab. Both functional and anatomic outcomes showed optimum results; VA improved compared to baseline, with statistical significance at 3 months, while CMT improved with statistical significance at all time points. In a subgroup analysis, there was no significant difference in male vs. female, while in an age-group analysis, the youngest age group showed a tendency of superior VA at all timepoints. In an analysis of naïve vs. non-naïve groups, the naïve group showed a superior result in both VA and CMT improvement, with statistically significant difference limited to the CMT at 1, 3 months. Within each group, the non-naïve group showed VA improvement despite lacking statistical significance, while the naïve group showed statistically significant improvement at 1,3,6 months. In both groups, the CMT improvement was evident at all time points with statistical significance. Only one case of adverse effect was identified (endophthalmitis). This showed faricimab’s effectiveness and durability in Korean patients in a real-world setting.

In a previous real-world study of faricimab treatment in 98 nAMD eyes that had suboptimal response to prior anti-VEGF therapy, CMT improved from 310 μm to 265 μm after four monthly injections of faricimab; the improvement of CMT was statistically significant at all time points, while the VA didn’t show statistically significant improvement^9^. Another study evaluating the real-world effectiveness of faricimab in 35 refractory nAMD patients, the BCVA (logMAR) improved from 0.84 ± 0.49 to 0.76 ± 0.49 (*p* = 0.025) at 3 months, while the CMT decreased from 447.65 ± 161.46 μm to 327.33 ± 147.78 μm (*p* < 0.001) at 3months^10^. In a real-world study involving 11 eyes (3 naïve eyes), BCVA improved from 0.612 ± 0.75 logMAR to 0.387 ± 0.54 logMAR, while CMT improved from 256.16 ± 12.98 μm to 245.43 ± 15.34 μm ^11^. The results of our study are comparable to these previous real-world studies, as there was a tendency of VA improvement over the entire study period, with statistical significance at 3months; CMT improved with statistical significance at all time points. Studies by Goodchild et al. ^9^ and Yufeng et al. ^10^ concentrated on the effectiveness on patients refractory to previous anti-VEGF treatment, which differs from our current study since 59 naïve eyes (20%) were included; however the subgroup analysis involving the non-naïve group (*n* = 234), which may be considered as patients refractory to previous anti-VEGF injections, also showed comparable results to previous studies, with marginal VA improvement and statistically significant CMT improvement. Moreover, an overall evaluation of both naïve and non-naïve patients, as done by Stanga et al. ^11,12,20^, provides a good representation of nAMD treatment in a real-world setting.

One important point to note is that while the CMT improved dramatically at 1 month after initiation of faricimab injection, VA only seemed to improve significantly after 3 months. This may imply that an anatomical improvement (i.e. decrease in CMT) doesn’t ensure a direct functional improvement (i.e. improvement in VA) – a similar tendency of preempt anatomical improvement and delayed functional improvement in nAMD patients treated with faricimab has also been identified in a previous study^21^. We suspect that inhibition of ANG-2 not only plays a role in anatomic improvement of CMT, but may also acts in functional improvement in a delayed or step by step fashion. Such a hypothesis should be validated upon future studies and with a larger sample size.

In this study, 85 eyes (29%) returned to another anti-VEGF treatment after suboptimal outcomes with faricimab. Already mentioned in our results, this suboptimal group showed difference in baseline characteristics, as they were predominantly non-naïve (*n* = 81, 95.3%) and had more anti-VEGF injections prior to faricimab injection. These results give a good representation of the difficulty in treating refractory nAMD in the real-world, despite the recent advances and growing variety in treatment choice of ani-VEGFs. Finding an optimum treatment strategy for these refractory cases, to not only maximize the functional and anatomical outcomes, but also to maximize the patient’s benefit in a cost-effective manner would be an important subject for analysis in future studies.

The subgroup analysis by age-group elucidates some implications in a perspective of health-care and social welfare. The youngest age-group (< 60 years old) showed superior VA outcome, which means that a patient group still within the working population can quickly get back to work after a successful or sustainable treatment of nAMD. This may contribute to a reduction in net loss of labor productivity, which may be important to an ageing population like that of Korea. To note, it has been estimated that a net loss of labor productivity (or indirect social costs) due to AMD in Korea was approximately 133 billion Korean wons (approximately $ 90,000,000), according to a study conducted in 2019 ^22^. This analysis needs to be further studied as the youngest age group (< 60 years old) only consisted 6% (*n* = 16) of the entire population, which may be the reason for the lack of statistically significant superiority in VA. The discrepancy between the superior VA and the poorer CMT outcomes in the 50s age group, may also have been due to a small sample size. One could argue that the CMT was thinner in the 50s age group at baseline (as shown in the results), and that they had more room for CMT aggravation than the other age groups. Analyzing the fluid status (intraretinal fluid, subretinal fluid) in these cases may give more perspective, along with future studies with a larger sample size and longer periods of follow up.

In previous real-world studies about the effectiveness of faricimab in AMD, some studies concentrated on refractory cases, (Goodchild et al. ^9^ and Yufeng et al. ^10^), while others included naïve eyes together in their study^11,12,20^. Muth et al. ^12^ and Khanani et al. ^20^ all showed limited VA and CMT improvement in the naïve group; the non-naïve group had a superior result. In our study, VA and CMT all improved with a higher magnitude in the naïve group, although not statistically significant. Within each group, it was evident that VA and CMT improvement was superior in the naïve group. This difference compared to previous studies may be due to the difference in the real-world cohorts given in each of the studies. Our study shows that not only is faricimab non-inferior in naïve patients, but they may be more effective in naïve patients. A possible assumption is that a bispecific inhibition of both VEGF-A and Ang-2 at the initial phase of neovascularization in nAMD may change the macular chorioretinal microenvironment such that it ultimately inhibits a rigorous progression into a more advanced nAMD. This would not only have impact in the anatomy of the patient but would significantly impact the visual prognosis.

Fluctation in CMT has shown to be inversely correlated with visual outcomes of nAMD patients being treated with anti-VEGF injections in previous studies^18,19^– the analyses on CMT fluctation in this study also showed similar results. 6 month VA was used as the outcome variable for multiple linear regression, because 3 month VA seemed relatively short of a time period, while 12 month VA lacked sufficient number of patients who reached such a follow-up period in this study. The multiple linear regression model depicted in Table 3 showed CMT fluctation (-0.04 [-0.07, 0.00] ETDRS letters of 6 months VA per CMT SD, 0.036) as a statistically significant predictor of VA at 6 months with an inverse correltation. The magnitude of associaton was also comparble to the study by Chen et al.^18^, where CMT standard deviation in 100s µm showed 12, 24 month VA estimates as -0.78 [-3.88, 2.31] and − 15.41 [-20.98, -9.83] respectively, which may translate to -0.0078 [-0.0388, 0.0231] and − 0.1541 [-0.2098, -0.0983] if the increments for CMT SD were considered as 1 μm, as done in this study. The mean difference of VA stratified in quartiles of CMT SD further helps visaulize the inverse correltation between VA and CMT fluctation (Fig. 9b.) – the more CMT fluctates, the poorer the visual prognosis seems to be. The relationship between CMT SD and the subgroups are also worth noting. Although gender, age, naïve vs. non-naïve, didn’t show statistically significant association with CMT SD, age showed a positive correlation with CMT SD (0.31 [-0.31 0.93] µm of CMT SD per years of patient’s age, *p* = 0.997) and non-naïve patients also showed positive relation with CMT SD (3.60 [-10.59, 17.79] µm of CMT SD compared to naïve patients, *p* = 0.618). This meant that naïve and younger patients tended to have lower fluctuation in CMT, with which we may infer better visual outcomes; this fits the subgroup analyses performed above, which suggested promising outcomes for the “young and naïve”.

There are several limitations to note in this study. Our study was a single-armed study with no control group, thus limiting the quality of our interpretations since other confounding factors may have contributed to the results. A comparative cohort may benefit the credibility of our findings in future studies. Secondly, a lack of a sufficiently large population limited our study. As shown in the VA results, although the mean VA improved compared to baseline at all timepoints, statistical significance was only noted at 3 months. An analysis with a larger population size may add statistical significance of VA at other timepoints. Also, a larger population size seems crucial in the subgroup analysis; our analysis that the youngest age group had a superior visual prognosis, was only supported by a sample size of 16 patients. Thirdly, it should be noted that younger patients tend to be more treatment-naïve and present less advanced cases, such as fibrovascular formation and geographic atrophy, which could confound the analyses. Analyzing the nature of the nAMD (e.g. type of choroidal neovascularization) or characterizing the fluid components of the OCTs have not been done in this study. It seems crucial that these classifications (e.g. by types of CNV, by the presence of intraretinal fluid, subretinal fluid, sub-RPE fluid, fibrosis, hemorrhage, atrophic change) be performed in future studies, in order to enhance the clinical relevance of the effectiveness of faricimab in nAMD in the real-world. Lastly, this study analyzed nAMD patients in a relatively short time period, with a mean follow-up time of approximately 6 months. Further long-term studies may be needed to see the effects of sustained treatment with faricimab.

Despite some limitations, this study has its values as it is the first study that evaluated the real-world effects of faricimab in nAMD patients in Korea, with a relatively big sample size and heterogenous group of patients from 8 different university hospitals. We hope that this study, along with future improvements, will help our understanding of treating nAMD patients in the future.

## Conclusion

In conclusion, the real-world data of faricimab treatment in nAMD patients of Korea show promising functional and anatomic outcomes. In a subgroup analysis, younger patients had a better visual prognosis, which gives promising expectations in reducing social costs by preventing a loss of labor productivity. Naïve patients treated with faricimab showed superior improvement in VA and CMT with marginal significance, indicating that treatment with faricimab at the initial stages of neovascularization may change the course of the nAMD and potentially improve visual prognosis. Lower CMT fluctuation was associated with better visual acuity, and had a correlation with younger age and naïve patients. With future improvements, we hope that this study will expand our understanding of treating AMD patients in the real-world.

## Data Availability

The datasets used and/or analyzed during the current study are available from the corresponding author on reasonable request.
